# Study Protocol: Does an Acute Intervention of High-Intensity Physical Exercise Followed by a Brain Training Video Game Have Immediate Effects on Brain Activity of Older People During Stroop Task in fMRI?—A Randomized Controlled Trial With Crossover Design

**DOI:** 10.3389/fnagi.2019.00260

**Published:** 2019-09-18

**Authors:** Robin Maximilian Himmelmeier, Rui Nouchi, Toshiki Saito, Dalila Burin, Jens Wiltfang, Ryuta Kawashima

**Affiliations:** ^1^Department of Functional Brain Image, Institute of Development, Aging and Cancer (IDAC), Tohoku University, Sendai, Japan; ^2^Department of Psychiatry and Psychotherapy, University Medical Center Göttingen (UMG), Göttingen, Germany; ^3^Smart Aging Research Center (SARC), Tohoku University, Sendai, Japan; ^4^German Center for Neurodegenerative Diseases (DZNE), Göttingen, Germany; ^5^iBiMED, Medical Sciences Department, University of Aveiro, Aveiro, Portugal

**Keywords:** acute benefit, brain training, combination training, high-intensity physical exercise, stroop, video game

## Abstract

**Background**: Elderly people are affected by processes leading to decline in various aspects of daily living that impair their quality of life. Regarding neurological aspects, executive functions have been shown to be valuable for daily life and to slow decline during aging. Most intervention studies intended to improve cognitive functions during aging specifically address long-term destructive processes and countermeasures. However, to an increasing degree, studies also investigate the acute benefits that prove to be useful for daily life, such as physical exercise or video games in the form of exercise video gaming (“exergaming”). Because little is known about the change in cognitive ability following acute intervention of a combination of physical exercise and video gaming, especially for older people, this work is designed as an attempt to address this matter.

**Methods**: This study is a randomized crossover controlled trial to test the response to an acute bout of high-intensity physical exercise followed by a short session with a brain training (Brain Age) video game in physically active and cognitively healthy older adults (60–70 years). The response is measured using Stroop task performance (cognitive task for executive function) and related brain activity assessed with functional magnetic resonance imaging (fMRI). The control conditions are low-intensity physical exercise and Tetris for video gaming.

**Discussion**: This study is intended to provide insight into the alteration of executive function and its related brain activity from an acute intervention with a combination of physical exercise and video gaming in older people. The protocol might not be implementable in daily life to improve cognitive abilities. However, the results can support future studies that investigate cognition and the combination of physical exercise and video gaming. Moreover, it can provide real-life implications.

**Trial registration**: This trial was registered in The University Hospital Medical Information Network Clinical Trials Registry (UMIN000033054). Registered 19 July 2018, https://upload.umin.ac.jp/cgi-open-bin/ctr/ctr_view.cgi?recptno=R000037687.

## Background

Elderly people experience processes leading to decline in various aspects of daily living that impair their quality of life. Regarding neurological aspects, the executive functions, which include aspects such as working memory, inhibition, and cognitive flexibility (Diamond, 2013), have been shown to be valuable for daily life. Nevertheless, they decline during aging. Executive functions are important for processes such as planning and at a later time for executing objectives successfully, and for using prospective memory (Hering et al., 2014; McDaniel et al., 2014). Prospective memory has been shown to be associated with and to decline with processes such as working memory, inhibition, and shifting, which are executive functions (Hering et al., 2014). Various positive attempts have been undertaken for improving or at least preserving executive functions in several ways, e.g., nutrition, physical training, and cognitive training (Kramer et al., 2003; Kueider et al., 2012; Johnson, 2013). Eventually, it might not be just one, but rather a combination of these options that will help to improve quality of life (Phillips, 2017). Most reported intervention studies that improve cognitive functions have emphasized long-term application of at least 1 month or longer (Brehmer et al., 2012; Cheng et al., 2012). However, lately, studies also increasingly investigate the acute benefits that prove to be useful for daily life (Roig et al., 2013), such as those related to physical exercise (Tomporowski, 2003) or video games in the form of exercise video gaming (“exergaming”; Monteiro-Junior et al., 2017). This work was undertaken to address this matter because little is known about the change in cognitive ability and its related brain activity using fMRI following an acute intervention of a combination of physical exercise and video gaming, especially for older people.

### Physical Exercise

Acute bouts of physical exercise can be implemented in daily life as part of a morning routine. They are already performed in Asian countries such as Japan (Yamashita et al., 2001). When examining physical exercise as an acute intervention, its benefits for cognitive abilities can be discussed intensively, such as whether moderate-intensity or high-intensity exercise is superior. For more details please see the “Discussion” section. Meta-analyses conducted by Chang et al. (2012) and Ludyga et al. (2016) reveal that, with regard to executive functions, older adults (Johnson et al., 2016) especially benefit from acute intervention with physical exercise and demonstrate that the benefit is independent of the current fitness level. For that reason, untrained people can receive cognitive benefits just as trained people do. This study uses a high-intensity physical exercise intervention and applies cognitive tests after a delay of approximately 20 min. This delay will be used to apply the video game intervention. The question of whether greater cognitive improvement is found after high-intensity physical exercise compared to low intensity should be answered. Moreover, we must ascertain whether this benefit can be enhanced by combining the intervention with brain training video gaming.

### Video Gaming

Video games elicit cognitive benefits (Granic et al., 2014), even in older adults (Zelinski and Reyes, 2009; Toril et al., 2014; Stanmore et al., 2017). Older adults are increasingly interested in playing video games (Zelinski and Reyes, 2009). They might have some potential for improving health-related issues, also in a clinical and rehabilitation framework (Primack et al., 2012; García-Betances et al., 2015). Nevertheless, the outcomes of video gaming for older adults are quite controversial. A meta-analysis from Toril et al. (2014) shows that cognitive functions of older adults benefit from video gaming in general, but it also revealed that executive functions do not reach significant levels. Other studies have revealed that the executive functions can be enhanced in older adults (Basak et al., 2008; Green et al., 2012; Strobach et al., 2012; Anguera et al., 2013). For a more detailed consideration please refer to the “Discussion” section. The usage of video games for acute interventions is on the rise, but many aspects remain to be answered (Guzmán and López-García, 2016; Monteiro-Junior et al., 2017). A salient question is: which game should be used for this purpose? First, it should have a beneficial effect on executive functions. Second, it should be conducted as an acute session to fit in the 20-min pause after the physical exercise. Third, the video game should be interesting also for older adults. One video game that fulfills all these requirements is Brain Age (Nintendo Co. Limited, Kyoto, Japan), also known as Dr. Kawashima’s Brain Training. It is a popular brain training series based on the scientific work of Prof. RK developed for handheld consoles from Nintendo (Kawashima et al., 2005; Uchida and Kawashima, 2008). It is meant to improve cognitive functions merely by playing a few minutes a day. Results demonstrated that common tasks among others such as simple arithmetic calculations and fast reading are beneficial for the executive functions (McDougall and House, 2012; Nouchi et al., 2012). Because Brain Age has only been conducted in a long-term setting with older adults (Nouchi et al., 2012), it is now to be examined whether it can also enhance the executive function in older adults in an acute intervention and whether there are additive effects if applied immediately after physical exercise.

### Combination of Physical Exercise and Video Gaming

For some people, physical exercise alone might be too boring to perform regularly. Combining it with video gaming in for example, “exergaming” (combination of physical exercise and video gaming), might improve compliance (Mat Rosly et al., 2017) and create engaging exercising programs for rehabilitation (e.g., decrease risk for falls; Hasselmann et al., 2015). Video games are meant to be fun; they excel at inducing the so-called flow (Sherry, 2004; Chen, 2007), which is a state of an individual when the challenge presented by a certain task matches the skill of this individual (Belchior et al., 2012). This state itself has beneficial effects on cognitive function (Shafer and Carbonara, 2015), which can be superior to usual cognitive training at least to some degree (Anguera and Gazzaley, 2015; Shute et al., 2015). Because exergaming is fun and emerging (Maillot et al., 2012; Verheijden Klompstra et al., 2014; Eggenberger et al., 2015; Lyons, 2015), it has a more positive influence on compliance than those of other rehabilitation methods. It “may be an effective way to promote physical and cognitive improvements among older adults” (Maillot et al., 2012).

One issue of studies investigating exergaming is that it is difficult to measure the extent of physical activity during such activities: do they really “exercise” or are they too passive? The approach such as that used in this study protocol to use physical exercise and video gaming separately but sequentially gives the opportunity to combine different conditions mutually. Consequently, it is distinguishable between the attribution of the different conditions to the improvement of the cognitive ability or the change in brain activity when measured in fMRI.

Because there might be different mechanisms affecting how physical exercise and video gaming respectively improve cognitive abilities (for additional details, see “Discussion” section), apart from the behavioral improvement at the cognitive test, differences in brain activity following different interventions will be recorded using functional magnetic resonance imaging (fMRI).

## Brain Activity in Elderly People During Physical Exercise and Video Gaming

This section specifically addresses what is known about brain activity in older adults with respect to cognitive tasks in general and after an acute intervention with physical exercise or with video gaming.

### Cognitive Task for Executive Function

For this study, the widely used Stroop interference task (Stroop, 1935) is used as the representative for the inhibition component of the executive functions (Miyake et al., 2000; Diamond, 2013). For simplicity, the color-word Stroop interference task will be henceforth designated as the Stroop task in further discussion. The participant is presented with color words such as green, red or yellow in either the same color as the word (congruent condition) or in a different color (incongruent condition), along with a second word in a neutral ink. The participant should compare the color of the first word with the meaning of the second word as quickly as possible. Usually for the incongruent condition, the reaction time is longer because the participant must inhibit the salient, but with different meaning of the word, the participant must instead must say the color in which the word is written. The Stroop task emphasizes the inhibition component of the executive function, but it is also influenced by visual spatial ability, language, or processing speed (Suchy, 2009;Diamond, 2013).

### Neural Correlates for Stroop Task in Elderly People

For older adults, neural substrates of the Stroop task using fMRI have usually been bilateral activations, especially of the anterior cingulate cortex (ACC) and lateral and medial frontal areas such as the left lateral prefrontal cortex (LPFC; Langenecker et al., 2004; Huang et al., 2012); the left inferior gyrus is particularly important for performance on the Stroop task (Langenecker et al., 2004). Prakash et al. (2009) compared differences in activation patterns of younger and older adults as the difficulty increases in Stroop task. Increasing difficulty is usually coupled with an increase of activation by younger adults. In older adults, while showing already increased activation intensity at baseline, this increase is absent. This absence is especially true for the dorsolateral prefrontal cortex (DLPFC). The conclusion is that older adults are not flexible anymore and are not able to adapt to higher difficulties, which underscores the necessity for recruiting additional brain areas to compensate for the decline in performance.

### Brain Activity of Elderly People After Physical Exercise

Considering how the brain activity of older adults changes after physical exercise, few reports describe studies that have investigated its acute effects either without neuroimaging (Kamijo et al., 2009) or in younger adults with neuroimaging such as fMRI (Li et al., 2014) and functional near-infrared spectroscopy (fNIRS; Yanagisawa et al., 2010; Tempest et al., 2017). Hyodo et al. (2012) have conducted the only study investigating the acute effects of moderate-intensity physical exercise on Stroop task performance in older adults using fNIRS as neuroimaging. No report of the relevant literature describes an investigation of acute effects of physical exercise conducted with fMRI (Voss et al., 2011), except for a report by Li et al. (2014), who investigated the working memory of younger female adults using N-back task.

Hyodo et al. (2012) described activation of the bilateral DLPFC, ventrolateral prefrontal cortex (VLPFC), and frontopolar area (FPA): the same brain areas as those reported by Langenecker et al. (2004) for older adults at rest. Especially, the right frontopolar area (R-FPA) is the neural substrate of improved cognitive performance after an acute bout of moderate exercise in older adults. Results suggest that during aging, R-FPA compensated for deterioration of the left dorsolateral prefrontal cortex (L-DLPFC), which is usually activated after physical exercise in younger adults (Hyodo et al., 2012). R-FPA activation has also been observed during Stroop interference in fMRI studies (Milham et al., 2001; Zysset et al., 2001). The Stroop reaction time correlates inversely with the activation of R-FPA.

### Brain Activity of Elderly People After Video Gaming

Alteration of brain activity through cognitive training or video games is not well understood. The activation patterns for executive function tasks after cognitive training differ (Di et al., 2014). Some reports have described increased activation in the prefrontal cortex after intervention (Olesen et al., 2004; Westerberg and Klingberg, 2007), but others have described decreased activation (Babiloni et al., 2009; Del Percio et al., 2009; Wartenburger et al., 2009; Gobel et al., 2011). Behavioral improvement of Stroop task performance after video gaming, especially for Brain Age, has been shown in younger adults after a long-term intervention (Nouchi et al., 2013), but no study has examined the conduct of an acute session of Brain Age in older adults as well as its neuroimaging.

Taking what can be expected about the acute intervention regarding the brain activity together, it can be concluded that an acute session of high-intensity physical exercise in older adults possibly engenders increases in activation of DLPFC, VLPFC, and FPA, but R-FPA especially correlates with better performance at the Stroop task. For video gaming, it can only be assumed that an acute session can affect brain activation in a way that either optimizes processes or exploits resources leading respectively to a decrease or increase in brain activation (Di et al., 2014). Whether an acute session of video gaming also transfers to better performance at Stroop task, or not, remains unknown.

## Purpose

This intervention is intended to test the alteration of the performance at a cognitive task for executive function (Stroop task) as well as related brain activity using fMRI to an acute bout of high-intensity physical exercise followed by a short session with a brain training video game in physically active and cognitively healthy older adults. It will be tested using a crossover design, so every participant acts as their own reference.

Some experiments have been conducted to investigate the long-term effects of physical exercise and video gaming on brain activity, but related to the acute effects, especially for the combination, the available literature is scarce. For physical exercise, some reports describe acute effects. Studies assessing video gaming as an acute intervention have been conducted, but no report has described an investigation of the acute effects of one session of video gaming on cognitive function, using either behavioral data or fMRI data. This report is the first of a study providing insight into how the brain activity and also the performance at the Stroop task might change in older people after an acute session of a combination of physical exercise and video gaming. With the design of this study, it is expected to be possible to compare between the physical exercise and video gaming conditions and to distinguish between their contributions on the improvement of cognitive function. Results from this study might not be suitable for the direct usage in daily life, but they pave the way for future studies investigating the combination of physical exercise and video gaming and their implications in daily life or in a clinical setting.

## Hypothesis

### Primary Hypotheses

1. Acute intervention with high-intensity physical exercise leads to significantly shorter reaction time at the Stroop task compared to intervention with low-intensity physical exercise.
2. Acute intervention with the brain training video game Brain Age leads to significantly shorter reaction time at the Stroop task compared to intervention with the Tetris video game.
3. Because an acute intervention of physical exercise might have a stronger effect on cognitive function than video gaming (McMorris and Hale, 2012; Bherer, 2015), the order for the other interventions with regard to the reaction time at the Stroop task is from short to long reaction time: (1) High intensity + Brain Age; (2) High intensity + Tetris; (3) Low intensity + Brain Age; and (4) Low intensity + Tetris.
4. Acute intervention with high-intensity physical engenders stronger activations in DLPFC, VLPFC, and FPA compared to low-intensity physical exercise.
5. Activation of R-FPA correlates with the performance (inversely with reaction time) at the Stroop task.
6. Acute intervention with Brain Age video game leads to significantly different activation patterns from those obtained from intervention with Tetris video game, which also correlates with better performance at the Stroop task.

### Secondary Hypotheses

1. Expectations for improvement in various fields might differ in some regards (e.g., performance at cognitive task, performance at everyday tasks) between the experimental and the control task (high vs. low intensity or Brain Age vs. Tetris, respectively).
2. Expectations of participants for improvement at the cognitive task (for both, physical exercise and video game interventions) correlate with the Stroop task performance (inversely with reaction time).
3. Engagements of participants during experimental and control conditions (high vs. low intensity or Brain Age vs. Tetris, respectively) are not significantly different from each other.

## Methods

### Trial Design, Setting and Registration

**Figure 1:**
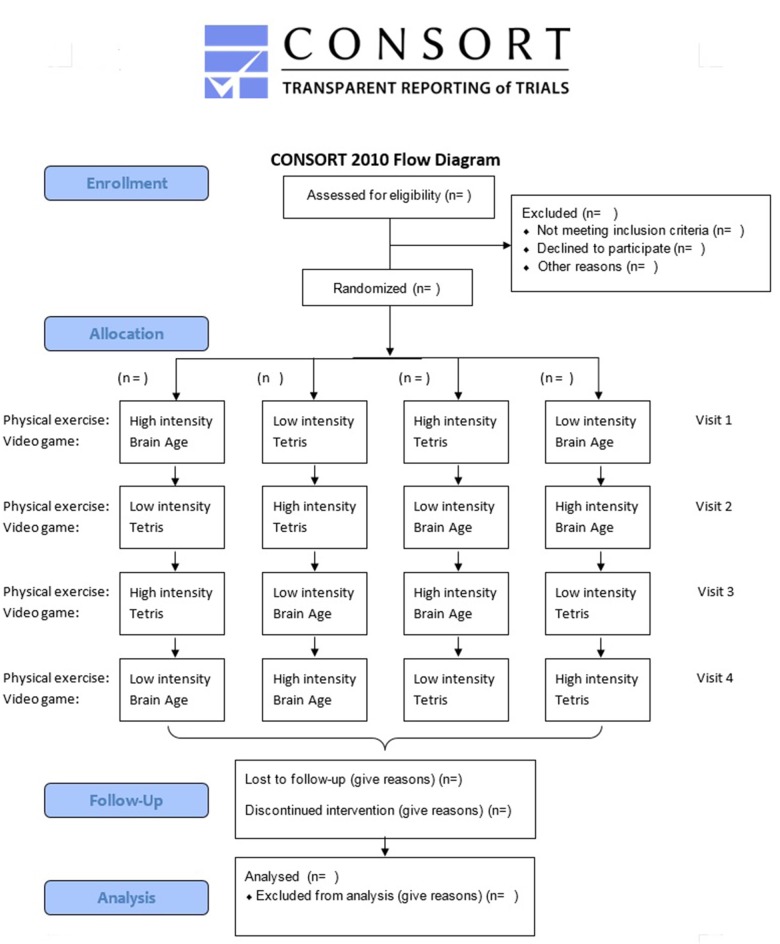
Consolidated standards of reporting trials (CONSORT) flowchart.

This study is a randomized crossover controlled trial to test the response of Stroop task performance (cognitive task for executive function) and the related brain activity using fMRI to an acute bout of high-intensity physical exercise followed by a short session with a brain training video game in physically active and cognitively healthy older adults (60–70 years). In four sequentially applied interventions, the participants will undergo every one of the four combinations of experimental and control conditions (Figure 1). Participants are blind to the study’s hypothesis. The Consolidated Standards of Reporting Trials (CONSORT) statement (Moher et al., 2010) as well as the Standard Protocol Items: recommendations for Interventional Trials (SPIRIT) statement (Chan et al., 2013) were used as a framework for developing the study methodology (see Supplementary Tables S1–S3). For CONSORT, the extension for within-subject design (Pandis et al., 2017) as well as the extension for non-pharmacological trials (Boutron et al., 2017) have been considered.

The study will be conducted at the Smart Ageing Research Center, Sendai City, Miyagi Prefecture, Japan. Each participant in this study will provide written informed consent before participating. The protocol of the study and the consent form were approved by the Ethics Committee of the Tohoku University Graduate School of Medicine. This study is registered with the University Hospital Medical Information Network (UMIN) Clinical Trial Registry (UMIN000033054).

### Participants

Participants will be recruited from the general population through an advertisement in the local newspaper. Interested participants will be invited to visit Tohoku University for a more detailed screening assessment.

### Eligibility Criteria

The numbers of female and male participants are expected to be the same. All included participants are expected to be 60–70 years old and reported as right-handed, native Japanese speakers, with normal or corrected-to-normal vision, normal color vision, not concerned about their own memory functions, not using medications known to interfere with cognitive function (including benzodiazepines, antidepressants, and other central nervous agents), not to have any magnetic resonance imaging (MRI) contraindication (e.g., metal implants, being pregnant), and having no disease known to affect the central nervous system, including thyroid disease, multiple sclerosis, Parkinson’s disease, stroke, severe hypertension or diabetes. To have the same baseline, all participants were unfamiliar with video gaming; all report playing less than 1 h of video games a week during the prior 2 years (Basak et al., 2008). To minimize the influence of subclinical degenerative conditions and because this study is intended to assess the effects of an acute bout of high-intensity exercise on cognitively active older adults, the following exclusion criteria are applied. First, to screen for cognitive impairment, the Mini Mental Status Exam (MMSE; Folstein et al., 1975) and the Frontal Assessment Battery at bedside (FAB; Dubois et al., 2000) are applied. The MMSE is a widely used screening tool for detection of cognitive impairments in older adults. It consists of 20 items. They measure the orientation for time and place, memory and attention, and language skills and visuospatial abilities. The score range goes from 0 to 30, with lower scores indicating a greater degree of general cognitive dysfunction. Those participants with a score of 26 (Nouchi et al., 2012) or lower at MMSE are excluded. The FAB evaluates the executive functions and consists of six subtests for similarities (conceptualization), lexical fluency (mental flexibility), motor series (programming), conflicting instructions (sensitivity to interference), go–no go (inhibitory control), and prehension behavior (environmental autonomy). The score range goes from 0 to 18, with lower scores indicating a greater degree of executive dysfunction. Those participants with a score of 12 or lower at FAB are excluded. Second, those who are not sufficiently physically active and who might have an increased health risk while performing a vigorous exercise are screened. For that reason, the International Physical Activity Questionnaire (IPAQ; Craig et al., 2003) and the new Physical Activity Readiness Questionnaire (PAR-Q+; Bredin et al., 2013) are applied. Among IPAQ of different types, for this study, the Short Form 7 Days Telephone (IPAQ-S7T) will be used. This script is a cost-effective tool to assess physical activity: the interviewer must read out loud and it consists of seven questions related to the time the participant spends on different activities during the last week. Based on the answers, the participants can be assigned to three physical categories: LOW, MODERATE, or HIGH. Although some studies question the validity of the questionnaire (Lee et al., 2011), it provides insights into the physical status of the participant. Participants that have a category of LOW at IPAQ are excluded. The PAR-Q+, a pre-participation screening tool for people who wish to participate in physical activity (Goodman et al., 2011), gives a rough evaluation of whether there are risks that must be assessed before starting the physical exercise. It is a four-page document that can be completed in approximately 5 min. The first page has seven questions related to the health and medical conditions of the participant which can be answered with YES or NO. If responses to one or more questions are YES, then the next pages present follow-up questions that more narrowly specify the health concern. Participants answering one of the first seven questions with YES at PAR-Q+ are excluded. Furthermore, participants with a resting blood pressure over 140/90 mm (Piepoli et al., 2016) and those who do not feel comfortable with exercising during the familiarization phase are excluded. To avoid including possibly cognitive impaired participants, those with a Stroop task correct answer rate of less than 80% in the analysis are excluded.

### Risks and Benefits to Participants

Each participant had been informed orally, assisted by the pre-exercise assessment document used for assessing the risks of the intervention in the following section at the first meeting in the course of the description of the study and before each session in the familiarization and intervention phase. They were required to give their consent and to sign the document before entering each session.

Regarding physical exercise, one could have concerns about performing high-intensity training in older adults because some risk exists of an acute cardiovascular event. Examples are cardiac dysrhythmia up until sudden cardiac arrest, sudden pulmonary vascular congestion, when the heart cannot keep up with the physical exercise, collapse with risk of injury while falling down, and acute myocardial infarction. Although it is true that the relative risk is higher during the physical exercise session compared to rest, the absolute risk is still very low (Riebe et al., 2015). In fact, a meta-analysis of high-intensity training even in patients with cardio-metabolic disease indicates that the risk is low and that it is safe to conduct (Weston et al., 2014). A review by Rognmo et al. (2012) that investigated the risk among coronary heart disease patients found in a total of 129,456 h during moderate-intensity training one fatal cardiac arrest and in 46,364 h of high-intensity training two nonfatal cardiac arrests, but no myocardial infarctions, thereby also finding low risk. Goodman et al. (2011) concluded: “*In conclusion, the mean rate of adverse events during exercise testing, including individuals referred for clinical exercise testing, based on our review article of the literature is less than 0.3 fatal events per 10,000 tests and 2.9 nonfatal events per 10,000 tests. Higher rates are observed for nonfatal events, although the adverse event reported in this context varies from mild arrhythmias that are not considered life threatening, to serious ventricular arrhythmias associated with increased risk of sudden death*.” Because only apparently healthy participants and not cardiovascular patients are recruited, the risk might be even lower. High-intensity training has already been conducted in older adults and has come to be regarded as safe (Hwang et al., 2016). As dangerous as the complications sound, severe incidents are often preceded by warning symptoms (Riebe et al., 2015). Examples are pain in the chest or in the legs that is unfamiliar while performing physical exercise [feeling of a stinger (“burning muscles”) in the legs can happen, but it is usually not harmful], chest tightness or discomfort, vertigo, feeling of fainting, and problems with breathing. If regarded seriously, these indicators can foreshadow difficulties and prevent major incidents. In conclusion, adverse acute events can happen during the familiarization and intervention phase, but the risk is very low and the described complications are rare. In addition, the participant might experience fatigue immediately after exercise, with muscle soreness on the days following the physical exercise.

During the video game session, the possibility of feeling fatigue exists, especially because of eye movements during video gaming, which are usually transient and which are not harmful.

Related to the fMRI, the possibility exists of feeling tired while performing the cognitive task. The participant might feel uncomfortable because of the loud background noise of the machine or the possible feeling of constriction because of the lack of space.

### Sample Size

Calculation of the sample size is not possible because no similar study has been reported to date. Soares et al. (2016) report that the optimal sample size for adequate reliability and power in fMRI studies might be between 16 and 32 participants. Zysset et al. (2007), who also conducted an experiment with Stroop task in fMRI, respectively used 23 and 24 participants per group. We also aim to include 24 participants.

### Interventions Overview

**Figure 2:**
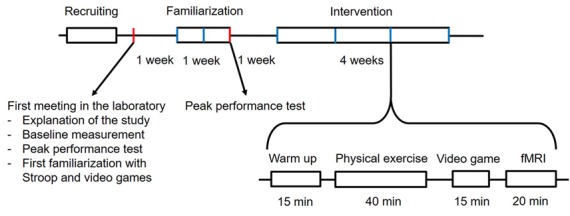
Timelines and phases of this study.

To check whether the participants can perform moderate to vigorous exercise and as already conducted in other studies (Knowles et al., 2015; Hwang et al., 2016; Grace et al., 2018), the participants will attend the familiarization phase over the course of 1 week and three sessions in all. Training at the first session is only performed at moderate intensity. The duration at high intensity is increased gradually for the second and third session. At the end of each session, the participants also practice the Stroop task and the video games for a total of 15 min. In the following intervention phase, each participant will sequentially perform four interventions with one intervention per week (four interventions after 4 weeks in all). The interventions are conducted once a week because reports of earlier studies state that to achieve a sufficient recovery in older adults, a break of at least 5 days is necessary between high-intensity physical exercise sessions (Herbert et al., 2015). Every intervention consists of a warm-up for the Stroop task to prevent the learning effect (Green et al., 2014). Each is followed by the actual physical exercise at either high or low intensity. After physical training, the participants go to the next room, where they can sit down and directly start the video gaming session with either the brain training video game “Brain Age” or with “Tetris.” The intervention is finished by the fMRI session using the Stroop task to investigate its related brain activity. Because there are two variables with two levels each, the possible combinations sum up to a sequence of four different interventions that one participant must undergo in a Latin square counterbalanced order (see Figures 1, 2). Each week, all participants will perform interventions one after another from morning until evening on two consecutive days. Participants are instructed to avoid strenuous physical activity during the 48 h (Knowles et al., 2015) preceding each intervention session. Each participant is also asked to refrain from food for 1.5 h and to have only a light meal beforehand, to refrain from caffeine and alcohol for 24 h (Knowles et al., 2015) before each intervention. Participants are asked to maintain their current lifestyle and not to make any changes to their level of physical activity or diet (Coetsee and Terblanche, 2017b).

### Physical Exercise Intervention

Participants perform physical exercise on an ergometer cycle (DK-8718RP; Daikou Corp., Japan). To tailor the intensity of the physical exercise for each, each participant performs the peak performance test at the first meeting and the last session of the familiarization phase to ascertain the maximal heart rate (HRmax). The intensity of the cycle ergometer has a range from level 1 until level 16. While cycling, the power is displayed in watts. In the beginning, the participant sits calmly on the cycle ergometer for 3 min and then starts to cycle. Initial mechanical power was set at level 1 for 2 min. the intensity was then increased by one level every 2 min until the limit of tolerance, with a fixed pedaling cadence of 60–70 revolutions per minute. The heart rate was displayed and recorded during each exercise session using a heart rate telemetry system (Polar H10 with app Polar Beat, ver. 2.5.2, on smartphone Freetel model FTJ17C00, Android ver. 7.1.2, retrieving data from online portal Polar Flow). At the end of every level, the participants rated their exhaustion on a Borg Rating of Perceived Exertion (RPE) Scale from 6 to 20 (Borg, 1982); the power will be recorded. Criteria used to end the exercise test stress were: (1) the inability to maintain the required pedaling cadence; (2) symptoms of fainting, vertigo, fatigue, dyspnea, or leg fatigue/pain; (3) achievement of age-predicted maximal heart rate, 208–0.7*age (Tanaka et al., 2001); and (4) rating of perceived exhaustion >18 on the Borg scale. The physical exercise is, if possible, always finished with a cool-down at lowest intensity for 3 min.

**Figure 3:**
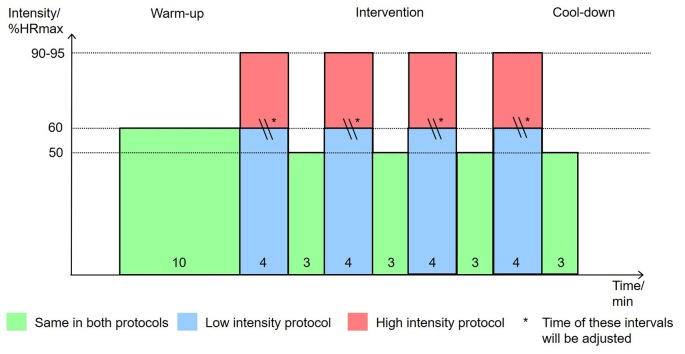
Physical exercise intervention protocol. Note: graph shows the time in minutes each participant spends at a specific intensity in percentage of their maximum heart rate.

According to earlier studies that also conducted high-intensity physical exercise with older adults (Hwang et al., 2016; Coetsee and Terblanche, 2017b) the protocol for the physical exercise in the intervention phase is the following: starting with a warm-up for 10 min at 60% maximal heart rate (%HRmax), the bout is followed by 4 × 4 min intervals at 90%HRmax for high intensity or at 60%HRmax for low intensity, respectively, interspersed by 3 × 3 min intervals at 50%HRmax for active recovery. During the recovery bout, the participants rate their exhaustion during the previous bout on a Borg RPE Scale from 6 to 20 (Borg, 1982). The bout ended with a cool-down for 3 min at 50%HRmax. The protocol of the low-intensity bout instead of is continuous because it has been conducted as an active control in most studies. The present study is designed to mimic the interval character of the high-intensity bout to increase the similarity of experimental and control condition. To match the caloric expenditure of the high-intensity and low bouts, the period of time of each interval at 60% peak heart rate in the low-intensity bout is prolonged based on calculations incorporating the power at 60% and 90% peak heart rate from the peak power test of each. The total exercise period is 38 min for high intensity and 38–54 min for low intensity. The participants were instructed to maintain a cadence of 60–70 rpm, which was checked by the examiner. To avoid risks that might harm the health of the participants, the following criteria were used to interrupt the exercise: (1) symptoms of fainting, vertigo, fatigue, dyspnea or leg fatigue/pain; (2) reaching age-predicted maximal heart rate, 208–0.7*age (Tanaka et al., 2001); and (3) rating of perceived exhaustion >18 on Borg Scale. The physical exercise is, if possible, always finished with a cool-down at lowest intensity for 3 min (see Figure 3).

### Video Game Intervention

For brain training video game intervention, Brain Age (Nintendo Co. Limited) has been used. To observe the proper results, we recommend the use of an active control group (Green and Bavelier, 2008). Tetris (Nintendo Co. Limited, Kyoto, Japan) has been shown as and has already been used as an appropriate active control to Brain Age (Nouchi et al., 2012, 2013) because it induces flow similarly to video games (Belchior et al., 2012), but it does not improve executive functions (Nouchi et al., 2013). Participants were instructed to play a commercial video game, either “Brain Age” or “Tetris,” for 15 min on a portable Nintendo Dual Screen (DS) × XL console. For Brain Age, the participants were required to play the Calculate × 100 and the Reading aloud games. Calculation × 100: the player must solve 100 simple arithmetic calculations as quickly as possible by writing the solution onto the touch screen of the Nintendo DS, Reading Aloud: participants read aloud excerpts from Japanese classical literature. Depending on the speed of the participants, they were asked to repeat from the beginning until finishing one run of the games. Tetris (Nintendo Co. Limited, Kyoto, Japan) is a 2D tile-matching puzzle video game. During the game geometric shapes composed of four blocks, each falling down one by one, and the player can move them to the side or rotate them while they are falling. The goal of the game is to keep the game going as long as possible. Therefore, the aim is to get a horizontal line of block with no gaps. The line disappears, providing more space and therefore more time. As the game progresses, the difficulty is increased by increasing the speed at which the geometric shapes fall down. The game ends when the player cannot manage to create full lines. Therefore, they can disappear, but the blocks pile up until the top of the screen. Tetris has been shown as and has already been used as an appropriate active control to Brain Age (Nouchi et al., 2012, 2013) because it does not improve executive functions. To familiarize the participants with the video games, they were practiced among the Stroop task at the first meeting and for 15 min in all after each session during a familiarization phase and before each session in the intervention phase.

### Cognitive Task

The color-word matching Stroop task was adopted and conducted in an event-related design (Yanagisawa et al., 2010; Hyodo et al., 2012) using software (E-Prime 2.0.10.42, E-Studio 2.0.10.147; Psychology Software Tools, Inc., Sharpsburg, PA, USA). The participant is presented with two rows of words and is instructed to decide whether the meaning of the color word in white ink in the bottom row matches the color of the word in the first row. The participants can input their choice by pressing a button with the index finger and the middle finger to give “yes” or “no” responses. The order in which the participants must answer “yes” or “no” is randomized. The assignment, whether the index finger is “yes” and the middle finger is “no” or vice versa, is counterbalanced across participants and across the two halves of one experimental session. The correct answer rate assigned to yes and no is 50% each. The correct answer rate and reaction time are also measured.

Trials of two types are compared in the Stroop task. For the congruent trials, the top row were words “RED,” “BLUE,” “YELLOW” or “WHITE” printed in their respective ink and the bottom row contains the words “RED,” “BLUE,” “YELLOW” or “WHITE” in black ink. For the incongruent trials, the top row includes the words “RED,” “BLUE,” “YELLOW” or “WHITE” in an incongruent color, whereas the bottom row is the same such as in the congruent trials. Each experimental session consists of two halves separated by a small break, each with 48 congruent and 48 incongruent trials that are presented in random order. Consequently, the total number is 192 trials (96 congruent and 96 incongruent trials). All word stimuli are presented in Japanese. To prevent the participants from particularly addressing the bottom row and blurring out the top row, the top row is presented 300 ms earlier than the bottom row. Visual attention should shift automatically to the top row then (Zysset et al., 2007; Hyodo et al., 2012). The stimulus is presented on the screen for 2 s. To avoid prediction of the timing of the subsequent trial, each trial is separated by an interstimulus interval showing a fixation cross for 2 (96 trials), 4 (72 trials) or 6 s (24 trials). The whole session lasts around 16.8 min plus the brief pause between the two halves.

As explained elsewhere (Klingberg, 2010; Green et al., 2014), to avoid learning effects, the Stroop task is practiced among the video games at the first meeting and for 10 min in all after each session during the familiarization phase and before each session in the intervention phase.

### fMRI Session

All MRI data are acquired with a Philips 3-T scanner. In the scanner, stimuli are presented visually through a MRI-compatible monitor (BOLDscreen 32 LCD for fMRI; Cambridge Research Systems). Participants have a four-button fiber optic response box with their right hand, which is used to record behavioral responses. To examine the tasks specifically, participants wear earplugs to reduce the scanner noise. Head motion is minimized using foam pads and a headband. First, functional images are acquired using echo-planar functional images (EPIs) sensitive to blood oxygenation level-dependent (BOLD) contrast (64 × 64 matrix, TR = 2,000 ms, TE = 30 ms, flip angle = 70°, FOV = 24 cm, 34 slices, 3.75 mm slice thickness). Anatomical scans begin by acquiring a T1-weighted sagittal localizer series.

## Outcome

The primary outcome measures for all participants are the reaction time and correct answer count from the Stroop task as well as its related brain activity using fMRI after each of the four interventions. Additionally, the secondary outcome measures will be recorded similarly to the scores from the questionnaires for engagement after each intervention, the expectation after the familiarization phase and after the intervention phase, age, and gender at the first meeting.

### Randomization

We conduct four interventions per participant and use a Latin square counterbalancing that sums up to four sequences in all (see CONSORT flowchart). First, it would be wasteful for organizational purposes and costs for MRI to cover every possible sequence (24 in total) because it would necessitate 24 separate intervention sessions for each participant. Second, the risk of having a bias because of carry-over effects might be higher when two high-intensity bouts follow each other than with two low-intensity bouts because results demonstrated that biomarker for cognitive improvement (e.g., brain-derived neurotrophic factor, BDNF) might still influence the performance, even after 7 days (Skriver et al., 2014). It is not confirmed, but by separating the two high-intensity bouts from each other, it might decrease this bias. To make it more homogeneous, high-intensity and low-intensity bouts are conducted in an alternating fashion.

### Allocation and Implementation

Allocation of the participants to one of four sequences is done at the end of the familiarization phase (1 week before intervention phase) through a computer-generated sequence using random allocation software Graph Pad’s QuickCalcs Random Mumbers^1^. Based on a randomly assigned number list, the participants are allocated to each sequence.

### Blinding

Because of the study design, blinding is not properly possible because it is a non-pharmacological within-subject design. The intervention is visible to both, the participants as well as to experimenters who conduct the intervention. To somehow address this issue according to CONSORT, extension for non-pharmacological trials (Boutron et al., 2017), first, the participants are blinded to the study’s hypothesis. Second, we assess the expectation as well as the engagement and take them into consideration for the analysis.

### Expectation and Engagement

Apart from the degree of physical exercise, another issue is that improvements in cognitive functions also depend on psychological factors such as one’s expectation about the outcome (Boot et al., 2013a) or engagement during the intervention (Green et al., 2014; Benzing et al., 2016). This study is designed to address these issues by considering the expectations and engagement of the participants. At the first meeting, the study protocol is explained to participants, followed by a practice session with the Stroop task and both video games. They are told that it should be investigated whether high-intensity or low-intensity training and whether Brain Age or Tetris has a stronger effect on Stroop task and brain activity. The participants are blinded to the expected outcome and are asked after the last session of the familiarization phase and after the last session of the intervention phase about their subjective feeling to what extent the interventions might affect cognition (Boot et al., 2013a). Results of earlier studies suggest that differences of subject feelings (e.g., enjoyment, motivation, fatigue, satisfaction) between the experimental (high-intensity physical exercise, Brain Age video game) and control (low-intensity physical exercise, Tetris video game) condition might affect the improvement of cognitive function (Green and Bavelier, 2008). Because Brain Age and Tetris have been shown to have equal levels of engagement, it is expected that no significant difference exists in engagement between Brain and Tetris. In case there is a difference, it will be checked whether the performance at the Stroop task as well as the brain activity in the fMRI correlate with the engagement score. Based on the suggestion, the participants are asked to respond to questionnaires related to their subjective feelings weekly after each intervention (Boot et al., 2013a; Nouchi et al., 2013). Participants rated both questionnaires using a Likert scale, with 1 representing strong disagreement and 7 representing strong agreement with the given statements.

### Stroop Task Analysis

We will use R (R Core Team, 2018) and lme4 (Bates et al., 2015) to perform linear mixed effects analysis of cognitive performance in dependence of physical exercise and video gaming. For reaction time at the Stroop task, we enter physical exercise intensity (high/low), video game (Brain Age/Tetris), week (1, 2, 3, 4), congruency of the Stroop task (congruent/incongruent), age (60–70 years), and gender (male/female) as fixed effects into the model. As random effects, we have intercepts for participants, as well as by-participant random slopes for the effect of physical exercise intensity and video game. Expectations for improvement at the cognitive task also enter as fixed effects into the model and the correlation with other predictors should be checked. For the correct answer count at the Stroop task, we will use the generalized linear mixed-effects model analysis and take the same fixed and random effects as above. Visual inspection of residual plots should be checked to ascertain whether they reveal any marked deviations from homoscedasticity or normality. *P*-values are obtained from likelihood ratio tests of the full model with the effect in question against the model without the effect in question.

### Analysis of Expectation and Engagement

We use the same linear mixed-effects analysis for the Stroop task analysis. For the rating of expectation and engagement, we enter physical exercise intensity (high/low), video game (Brain Age/Tetris), week (0, 1, 2, 3, 4), age (60–70 years), and gender (male/female) as fixed effects into the model. As random effects, we have intercepts for participants, as well as by-participant random slopes for the effect of physical exercise intensity and video game. Experimental and control condition are compared whether any significant differences exist in expectation and engagement between the experimental condition (high intensity, Brain Age) and the control condition (low intensity, Tetris) and for expectation whether the answers changed significantly before and after the intervention.

### Analysis of Physical and Game Performances

To check the exercise intensity in all conditions, we calculate average heart rates during the high and low-intensity exercise. We will check that the average heart rates will be around 90%HRmax for high intensity or at 60%HRmax for low intensity. In addition, we use the same linear mixed-effects model analysis for the expectation and engagement. We expect that the effect of the physical exercise intensity will be found.

For the game performance, we will separately calculate the average game scores of Brain Age and Tetris. For Brain Age, we will use the average time to solve 100 simple arithmetic calculations and read aloud sentence. For Tetris, we will use the average game scores. Then, we will conduct linear mixed-effects analysis for each game score separately. For the average time of Calculate × 100 and the Reading aloud games in Brain Age, we enter physical exercise intensity (high/low), age (60–70 years), week (1, 2, 3, 4), and gender (male/female) as fixed effects into the model. As random effects, we have intercepts for participants, as well as by-participant random slopes for the effect of physical exercise intensity. For the average score of Tetris, we will use the same model as the Brain Age analysis. We will check any significant difference in the exercise condition.

### fMRI Analysis

Functional imaging data preprocessing and analysis were performed with Statistical Parametric Mapping SPM12 (Wellcome Trust Centre for Neuroimaging, London, UK). In the preprocessing, all functional images were corrected for motions using realign, co-registered to T1-weight structural image, then spatially normalized into the Montreal Neurological Institute (MNI) template. These normalized images were spatially smoothed using a Gaussian kernel of 8-mm full width at half maximum (FWHM).

All preprocessed functional images were analyzed using two-step statistics at the subject level and the group level. In the subject-level (fixed-effect) analyses, experimental trials were divided into two conditions (congruent and incongruent). Then, brain activations associated with these conditions and six motion parameters (three translational and three rotational directions) were modeled by convolving a vector of task onsets with a canonical hemodynamic response function (HRF) in the context of the general linear model (GLM). Low-frequency components of fMRI time series were removed by high-pass filtering. For each subject, the incongruent trials were contrasted to the congruent trials. All contrasts yielded a *t*-statistic in each voxel.

The contrast images from the first-level analysis were used for the second-level analysis (Random Effect). We analyzed the data using a 2 × 2 repeated measures analysis of variance (ANOVA) to assess the main effects of A (physical exercise: high intensity vs. low intensity), B (video game: Brain Age vs. Tetris), and the A × B interaction using the flexible factorial approach in SPM12. We used a threshold of *P*-value using whole-brain cluster family-wise error (FWE) correction at *P* < 0.05, with an uncorrected voxel-level cluster-defining threshold of *P* < 0.001.

### Data Management

Personal information and data for all experiments are handled by the personal information manager director of Tohoku University. Access to information requires a key that is securely kept and has limited access. These internal checks and balances ensure the security of all data and personal information. After the experimental period ends, the data will be consolidated. Any information linking data back to the participant will be discarded to ensure that the data are truly anonymous. Data destruction will not be conducted.

### Data Monitoring and Auditing

We will follow the recommended guidelines for clinical research in Japan^2^. According to the guidelines, data monitoring by a third party is unnecessary for this study because we are not providing participants with any medications, nor are we conducting surgery.

### Research Ethics Approval and Informed Consent

The Ethics Committee of Tohoku University Graduate School of Medicine approved the study protocol. They will approve the consent form. Participants will be given both an oral explanation and a written file that documents details of the study. Upon understanding, the participant will be asked to give written consent by signing the consent form. Participants might choose to withdraw from the study at any point in time. Should they choose to do so, their data would be destroyed.

### Confidentiality

All participants will be given subject identification numbers to which all the data will be linked. The file records that connect each participant to their identification number will be securely kept on laboratory servers during the study. After the study is concluded, the file will be kept for 3 years and will be destroyed afterward. This study is conducted using the operating grants of the group of Tohoku University of implementation officer.

### Declaration of Interests

For the practice of the present research program, no relation exists between the particular companies. In other words, no author had any conflict of interest related to this research project.

### Access to Data

As described previously, all personal information and data for the experiment are handled by the personal information manager director of Tohoku University. Access to information requires a securely kept key with limited access. These internal checks and balances ensure the security of all data and personal information.

### Dissemination Policy

Results of the study will be published formally in a journal. Participants, the public and other relevant groups will not be individually notified of the results. There is no publication restriction to note. Authorship of the study belongs to those specifically listed; no professional writer was involved. Currently, no plan exists to grant public access to the full protocol, participant-level dataset, and statistical code. For interested researchers, we have the following ideas for data sharing. For the study protocol, the current protocol article should be the full protocol. For statistical analysis, we will use open source softs and packages such as R and SPM for behavior and brain imaging analyses. We will not have any plans to use customized statistical codes. For the participants-level dataset, behavioral data will be available on Open Science Framework^3^ or will be uploaded the journal web as a supplemental information material. On the other hands, we will not share structural and functional MRI dataset. The MRI datasets will be available on requests after getting an approval of the reuse of MRI dataset from our ethical committee.

## Discussion

This study is a randomized within-subject crossover controlled trial to test the response of Stroop task performance (cognitive task for executive function) and the related brain activity using fMRI to an acute bout of high-intensity physical exercise followed by a short session with a brain training video game in physically active and cognitively healthy older adults (60–70 years). The control conditions are low intensity for physical exercise and Tetris for video gaming.

### Physical Exercise

As mentioned in the introduction, there are several variables to consider when using physical exercise as an acute intervention in order to improve cognitive abilities. To construe the outcome of acute exercise, certain variables to keep in mind include the exercise intensity (low, moderate or high), the time point at which the cognitive test is applied after the physical exercise (during, immediately after or after a brief pause), and the type of cognitive test (attention, executive functions, memory, etc.; Chang et al., 2012). Using these variables, hypotheses such as the inverted-U-shaped hypothesis (Chmura et al., 1994; McMorris et al., 2011), which predicts stronger benefits at moderate intensity than at low or high intensity, or the drive hypothesis (Chang et al., 2012), which posits higher intensity as producing better cognitive improvement, are explainable to a certain extent (Chang et al., 2012). Whereas the cognitive test is applied immediately after exercise, lower and moderate intensity are apparently more favorable for cognitive improvement than high intensity, which supports the inverted-U-shaped hypothesis. When cognitive testing is applied after a short delay from several minutes up to a few hours after the exercise, lower intensities exhibit no significant improvement. The degree of improvement shifts to higher intensities, which favors the drive hypothesis. Cognitive improvement and metabolic response seem to have their maximum effects at a delay of 11–20 min after high-intensity exercise (Chang et al., 2012). Results demonstrated that the speed at executive function tasks improves significantly after acute exercise, whereas the accuracy remains non-significant or even decreases slightly (McMorris et al., 2011; McMorris and Hale, 2012).

The mechanism behind the improvement of cognitive function after physical exercise is already widely debated. Especially after an acute intervention, it is most likely attributable to the release of neurotransmitters (Hillman et al., 2008; Skriver et al., 2014) such as BDNF (Piepmeier and Etnier, 2015), vascular endothelial growth factor (VEGF) or insulin-like growth factor 1 (IGF-1), catecholamines (McMorris and Hale, 2015), changes of cerebral blood flow (Ogoh et al., 2014), cerebral oxygenation and oxygen usage (Coetsee and Terblanche, 2017a), or arousal level (Kamijo et al., 2004). In young adults, it might be demonstrated that BDNF peaks after 15–20 min (Schmidt-Kassow et al., 2012) and right at the time when participants enter the fMRI session, which is also a reason why the physical exercise is conducted before the video gaming session.

### Video Gaming

Interventions with cognitive training or video games always aim to exert a so-called transfer effect (Hertzog et al., 2008). Transfer means to convert individual improvements, say, from a video game, to other cognitive tasks. The transfer can be divided further into near transfer, by which trainees improve on tasks that are not identical, but which are closely related to the training; with far transfer, performance is improved on non-related tasks or even on tasks in real-life situations. Video games show some difficulties in eliciting transfer effects (Green and Bavelier, 2008), even exhibiting weak to no correlation between video game experience and cognitive function (Redick et al., 2017). Nevertheless, in recent years, video games, especially action video games, have shown positive results on far transfer effects in areas such as visual spatial ability (Green and Bavelier, 2007; Green et al., 2010). However, other categories of video games show promising results in the same (Toril et al., 2016) or other cognitive functions as well (Basak et al., 2008; Oei and Patterson, 2013). Improvements in cognition related to video games tend to be shared features between the video games and real-world/cognitive task rather than generalized, non-specific transfer (Oei and Patterson, 2014). The mechanisms underlying cognitive improvement because of video games are yet to be elucidated. However, factors such as virtual environments, challenges, and reward systems might affect cognitive function (Monteiro-Junior et al., 2016). Thus, video games might present the difficulty to transfer to other cognitive challenges (Redick et al., 2017), so it can also turn out that Brain Age shows no positive results in this scenario where it is used as an acute intervention with an executive function task for older adults as participants (Ackerman et al., 2010; Owen et al., 2010). Nevertheless, it can be ascertained whether differences exist in brain activities between the conditions that might explain these circumstances.

### Combination of Physical Exercise and Video Gaming

For the combination of physical exercise and video games, animal experiments already demonstrate that there are additive improvements of cognitive functions with a combination of physical exercise and cognitive challenges (Fabel et al., 2009; Kempermann et al., 2010), but there is still a lack of data related to this topic of a combination of physical exercise and video gaming in general (Mishra et al., 2012). Some reports of studies of exergaming already exist, but the results they describe remain controversial (Ordnung et al., 2017; Stanmore et al., 2017). In older adults, dancing with a dual-task character (physical exercise and cognitive challenge) is superior on acute improvement task-switching reaction time (executive function) than comparable physical exercise alone (Kimura and Hozumi, 2012). Other studies have found no significant improvement after one session of exergaming compared to the control group (Guzmán and López-García, 2016; Monteiro-Junior et al., 2017), but have also found a reduced rate of perceived exhaustion (RPE) compared to moderate aerobic physical exercise alone (Guzmán and López-García, 2016). A recent systematic review from Zeng et al. (2017) concluded that results for exergaming used as a therapeutic tool are inconsistent, but there are no negative outcomes, so it might feasible to use for older adults (Chao et al., 2015), even with mild cognitive impairments (Hughes et al., 2014).

Because reports of the literature are scarce for many aspects of this study, e.g., acute intervention of video gaming for results at the Stroop task or for brain imaging in older adults, definite hypotheses are challenging to formulate. However, based on the remaining literature, this study should find answers to following hypotheses: an acute intervention with physical exercise might have a stronger effect on cognition than an acute intervention with video gaming (McMorris and Hale, 2012; Bherer, 2015). High-intensity physical exercise condition and Brain Age video game condition might show a better improvement in reaction time at the Stroop task than their respective control condition, low intensity and Tetris, respectively. Regarding the brain activation in older adults, acute intervention with high-intensity physical engenders increases in activation of DLPFC, VLPFC, and FPA compared to low-intensity physical exercise. Activation of R-FPA correlates with the performance (inversely with reaction time) at the Stroop task. For video gaming, this might be the first study to reveal differences in brain activity after an acute intervention with Brain Age or Tetris.

This study is designed to yield insight into the alteration of executive function and its related brain activity because of an acute intervention with a combination of physical exercise and video gaming in older people. The protocol might not be implementable in daily life to improve cognitive abilities. However, the results can support future studies that investigate cognition and the combination of physical exercise and video gaming as well as implications for real life.

## Strength and Limitations

This study combines the following aspects: an acute intervention with a combination of physical exercise and video gaming in older adults while imaging the brain activity with fMRI. Many studies of physical exercise and video gaming are conducted on a long-term basis. Studies that have investigated the acute effects of video gaming on cognition, especially with older adults and brain imaging, are still few to date. For example, acute intervention of physical exercise and brain imaging with fMRI has been conducted only with younger people (Li et al., 2014). Acute intervention of physical exercise and older adults have been conducted using only fNIRS as brain imaging (Hyodo et al., 2012).

Randomized within-subject crossover controlled trial with a 2 × 2 design where the physical exercise condition and the video gaming condition are conducted one after another makes it easy to compare and distinguish between the relative influence each condition has on the improvement of the cognitive abilities or difference in brain activity.

Especially regarding video gaming, an active control is included. The expectations and engagement of participants are considered, which has been recommended elsewhere (Boot et al., 2013b).

Some issues might arise because of the lack of blinding. To address this issue somehow, expectations and engagement are assessed. The assessments can be checked to evaluate correlation with performance at the cognitive test.

To improve the safety of the older participants during the screening phase, instead of using different tests for physical (IPAQ, PAR-Q+) and neurological conditions (MMSE, FAB), it would be more advisable to conduct proper medical examinations, particularly addressing participants’ physical (e.g., exercise electrocardiogram, lung function test) and neurological condition (orientating examination). A medical examination might also improve reproducibility because MRI scans can be examined by a neurologist to elucidate neurological or vascular pathologies (D’Esposito et al., 2003). Medical supervision during the intervention (Hwang et al., 2016) would further improve the participants’ safety.

Regarding physical exercise at high intensity, the possibility exists of a carry-over effect because of an overly short break. Despite having a 1 week break between interventions (2 weeks between high-intensity bouts), the performance of the following interventions might be affected, even after one high-intensity bout (Skriver et al., 2014). It is not confirmed, but it must be considered.

For application of the physical exercise, the intensity is commonly set relative to the maximal oxygen uptake (VO_2_max) of a participant (Carter et al., 2002; Burnley and Jones, 2007). For this study, heart rates have been used which should be sufficiently feasible, but it would probably be more accurate to use a spirometer for VO_2_ measurements. Furthermore, the cycle ergometer only has the option to set intensity with regard to levels of 1–16, which might be too rough. To allow for more fine-tuning, setting the intensity with respect to the power in watts is recommended.

## Generalizability

Currently, it is planned to conduct this study with participants in the region around Sendai, Japan. Consequently, these results can only be applied for a very specific population of very healthy Japanese people between 60 and 70 years of age. It would be advisable to repeat this study with participants of different types to infer results for a more general population, e.g., young people, people with vascular or cognitively impaired patients, people from different countries.

## Ethics Statement

The Institutional Review Board of the Tohoku University Graduate School of Medicine (Ref. 2018-1-106) provided ethical approval. Based on the Declaration of Helsinki, written informed consent will be received from each participant.

## Author Contributions

RH, RN and RK designed and developed the study protocol. RH and RN searched the literature, selected cognitive function measures, and created manuals to conduct and rate cognitive measures; contributed equally to this work; wrote the manuscript with TS, DB, JW and RK. RK also gave advice related to the study protocol. All authors read and approved the final manuscript.

## Conflict of Interest

RK is the creator of Brain Age. Tohoku University, to which RK belongs, has received royalties generated by Brain Age sales. RK has no other competing interest. This does not alter the authors’ adherence to general research policies on sharing data and materials. The remaining authors declare that the research was conducted in the absence of any commercial or financial relationships that could be construed as a potential conflict of interest.

## Footnotes

^1^https://www.graphpad.com/quickcalcs/randMenu/

^2^http://www.mhlw.go.jp/topics/bukyoku/seisaku/kojin/dl/161228rinsyou.pdf

^3^https://osf.io

## Supplementary Material

TABLE S1 CONSORT 2010 checklist.

TABLE S2 SPIRIT 2013 checklist.

TABLE S3 Table of SPIRIT 2013 checklist.

## Abbreviations

ACC: anterior cingulate cortex
BDNF: brain-derived neurotrophic factor
BOLD: blood oxygenation level-dependent
CONSORT: Consolidated Standards of Reporting Trials
DLPFC: dorsolateral prefrontal cortex
EPI: echo-planar functional image
Exergaming: exercise video gaming
FAB: Frontal Assessment Battery at bedside
FEW: family wise error
fMRI: functional magnetic resonance imaging
fNIRS: functional near-infrared spectroscopy
FPA: frontopolar area
FWHM: full width at half maximum
GLM: general linear model
HRF: hemodynamic response function
IGF-1: insulin-like growth factor 1
IPAQ: International Physical Activity Questionnaire
L-DLPFC: left dorsolateral prefrontal cortex
LPFC: lateral prefrontal cortex
MMSE: Mini Mental Status Exam
MNI: Montreal Neurological Institute
PAR-Q+: new Physical Activity Readiness Questionnaire
R-FPA: right frontopolar area
RPE: Borg Rating of Perceived Exertion
SPIRIT: Standard Protocol Items: Recommendations for Interventional Trials
TR: repetition time in functional magnetic resonance imaging
TE: echo time in functional magnetic resonance imaging
UMIN: University Hospital Medical Information Network
VEGF: vascular endothelial growth factor
VLPFC: ventrolateral prefrontal cortex
VO_2_max: maximal oxygen uptake
%HRmax: percentage of maximal heart rate.
